# Root architecture, rooting profiles and physiological responses of potential slope plants grown on acidic soil

**DOI:** 10.7717/peerj.9595

**Published:** 2020-08-24

**Authors:** Deivaseeno Dorairaj, Muhammad Fahmi Suradi, Nursyamimi Syafiqah Mansor, Normaniza Osman

**Affiliations:** Institute of Biological Sciences, Faculty of Science, Universiti Malaya, Kuala Lumpur, Malaysia

**Keywords:** Acid soil, *Hibiscus rosa-sinensis*, *Melastoma malabathricum*, Root profile, Slope species, Slope stabilization, *Syzygium campanulatum*

## Abstract

Globally, there has been an increase in the frequency of landslides which is the result of slope failures. The combination of high intensity rainfall and high temperature resulted in the formation of acidic soil which is detrimental to the healthy growth of plants. Proper plant coverage on slopes is a prerequisite to mitigate and rehabilitate the soil. However, not all plant species are able to grow in marginal land. Thus, this study was undertaken to find a suitable slope plant species. We aimed to evaluate the effect of different soil pH on root profiles and growth of three different potential slope plant species namely, *Melastoma malabathricum*, *Hibiscus rosa-sinensis* and *Syzygium campanulatum*. *M. malabathricum* showed the highest tolerance to acidic soil as it recorded the highest plant height and photosynthetic rate. The root systems of *M. malabathricum*, *H. rosa-sinensis* and *S. campanulatum* were identified as M, VH- and R-types, respectively. The study proposed *M. malabathricum* which possessed dense and shallow roots to be planted at the toe or top of the slope while *H. rosa-sinensis* and *S. campanulatum* to be planted in the middle of a slope. *S. campanulatum* consistently recorded high root length and root length density across all three types of soil pH while *M. malabathricum* showed progressive increase in length as the soil pH increased. The root average diameter and root volume of *M. malabathricum* outperformed the other two plant species irrespective of soil pH. In terms of biomass, *M. malabathricum* exhibited the highest root and shoot dry weights followed by *S. campanulatum*. Thus, we propose *M. malabathricum* to be planted on slopes as a form of soil rehabilitation. The plant species displayed denser rooting, hence a stronger root anchorage that can hold the soil particles together which will be beneficial for slope stabilization.

## Introduction

Acid sulphate soil that is frequently equated to soil infertility occupies land area of almost 50 million ha worldwide, including Southeast Asia, Australia, West Africa and Scandinavia (Ljung et al., 2009). It is estimated that approximately 30% of the world’s total land area consists of acid soils while over half of the world’s potential arable lands are acidic (Von Uexküll & Mutert, 1995). According to Sumner & Noble (2003), topsoil acidity (pH < 5.5) affects around 30 and 75% of the total ice free land area of the world and subsoil acidity, respectively. As for the Asian region, acid sulphate soils occupy 7.5 million ha and 112 thousand ha of land in Southeast Asia and China, respectively (Shamshuddin et al., 2014). In Malaysia, these kinds of soils were estimated to cover about 0.5 million ha area with 110,000 ha in the Peninsular (Poon & Bloomfield, 1977).

As for the tropics including Malaysia, the combination of high rainfall and ambient temperature causes intense chemical weathering and formation of thick soil profiles. Due to high rainfall, the soils of the tropics, Oxisols and Ultisols, are highly weathered, leached, acidic and low in base saturation (Foy, 1992) whereas Histosols, contain high levels of organic matter and very low mineral contents (Snyder, Jones & Gascho, 1986). In addition, excessive use of ammonium-based fertilizers as part of agriculture intensification and cropping practice had led to soil acidification.

Apart from leading to soil acidity, the annual rainfall of about 2,500 mm accelerates the rate of erosion which subsequently leads to landslide (Shafie, 2009). In addition, over the last decade, rapid land clearing of hillsides for the purpose of development and infrastructure expansion had created overburdened loads on the slopes thus increasing the frequency of slope failures (Szabo , 2003). Moreover, deforestation in the country has adverse effects on the hydrological cycle, particularly relating to increase in runoff and erosion (Pradhan et al., 2012). Plants play crucial role in rehabilitating landslide affected areas. However, most slopes in Malaysia are arid and infertile due to the lack of clay activities and buffering capacity hence reducing the survival rate and growth of potential slope plants. As the soil lacks water holding capacity, it leads to rapid percolation of water which subsequently leads to nutrient leaching such as the basic elements of calcium, magnesium, potassium and sodium (Lehmann & Schroth, 2003) resulting in the slope soil becoming acidic.

Moreover, the presence of soluble forms of Al, Fe and Mn, nitrites and various toxic organic acids in the soil is detrimental to plant productivity. Aluminium (Al) which is the third most abundant in the earth’s crust is present in soils in a variety of forms and is bound to the soil constituents, particularly clay particles and organic matter. As the soil pH drops or when the soil becomes acidic, Al is solubilised into soil solution, thus exposing the plants to Al toxicity (Kochian, 1995). According to Sanchez & Logan (1992), one third of the tropics, or 1.7 billion hectares, is acid enough for soluble aluminum to be toxic for most crop plants.

Correspondingly, the presence of excessive Al ions in the soil will hinder the growth and development of major crops, namely, soybean, wheat and maize (Kochian, Hoekenga & Piñeros, 2004). In addition, the ionic forms of Al reportedly inhibits root growth (Kopittke et al., 2015) and root elongation by destroying the cell structure of the root apex and thus affecting water and nutrient uptake by the roots (Zheng, 2010), and disturbs root functions, such as plasma membrane permeability (Ishikawa & Wagatsuma, 1998), lipid peroxidation (Ikegawa, Yamamoto & Matsumoto, 2000) and cell wall rigidity (Wehr, Menzies & Blamey, 2004).

Hence, a suitable and favourable erosion control ground cover is essential for long term stabilization of slopes. The use of vegetation or bioengineering technique is the right way forward for it is cheap, provides a quick fix to impeding problem, sustainable and is environmentally friendly. Plants play a crucial role in reducing moisture content of soil through evapotranspiration which allows the soil to absorb more water. This process facilitates reduction of pore pressure while increasing the shear strength of soil, thus increasing its resistance (Faisal & Normaniza, 2008).

Often, the slopes identified for rehabilitation are on acidic soils that contain excess aluminium (Al) ions which retards the growth and development of major crops. Thus, not all plants are suited to be planted in acidic soils. Nevertheless, many native plant species can establish itself in an extremely low soil pH (<3.5) (Osaki et al., 1998a; Osaki et al., 1998b) and are able to take up higher concentrations of Al in both roots and shoots (Watanabe & Osaki, 2002) such as *Melastoma malabathricum* L. (rhododendron) which is known as ‘Al accumulator’. Al accumulators are known to form stable complexes with organic and inorganic ligands by removing ionic Al from the soil; hence they thrive and survive in such soils (Watanabe & Osaki, 2002).

Furthermore, it was proven by Saifuddin et al. (2016) that this species exhibited the highest increment of soil pH and has the ability to rehabilitate the acidic condition of the slope. Hence, it is crucial to understand the process of the plant development on slope areas. Besides *M. malabathricum*, *Hibiscus rosa-sinensis* (hibiscus), an ornamental woody plant is a potential phytoremediation plant for it is a contaminant accumulator such as cadmium (Cd) and lead (Pb) (Bhaduri & Fulekar, 2012). In addition, *Syzygium campanulatum* (redbud) has the ability to withstand soil infertility and can increase its biomass (Arunbabu et al., 2015).

The plant root system and architecture play a fundamental role in providing mechanical support against wind, snow and gravitational forces exerted by the plants while binding the soil particles together. In other words, the manifestation of slope stability by vegetation is due to the strong anchorage provided by roots and rhizomes that hold the soil particles, thus increasing the rate of infiltration which further decreases soil run-off (Pitt et al., 2003). According to Faisal & Normaniza (2008), the weight of vegetation adds to the load which is the driving force while the roots reinforces the soil thus increasing the resisting force.

Thus, in this study we aimed to evaluate the effect of different soil pH on root profiles and growth of three different potential slope plant species namely, *M. malabathricum*, *Hibiscus rosa-sinensis* and *Syzygium campanulatum*. The main objective of this experiment is to screen for a suitable plant that could be grown on slopes with acidic soil.

## Materials & Methods

### Site description and experimental design

The plants were grown in a glasshouse at Rimba Ilmu, Institute of Biological Sciences, University of Malaya, Kuala Lumpur, Malaysia (3°7′52.1076″N, 101°39′25.218″E). The Photosynthetically Active Radiation (PAR), relative humidity (RH) and atmospheric temperature of the glasshouse ranged at 300–2,000 µE mol m^−2^ s^−1^, 65–90% and 25−28 °C, respectively. The selected plants were *Melastoma malabathricum, Hibiscus rosa-sinensis* and *Syzygium campanulatum.* Each species was planted at three different soil pH levels; 3-4(T1), 4-5(T2) and 6-7(T3). The plants were grown in polybags with a size of 36 cm × 20 cm × 16.5 cm, and a soil volume of 8,910 cm^3^. The soils of T1 and T3 were pre-treated with aluminium sulphate (Al_2_(SO_4_)_3_) to obtain the three pH levels. A soil incubation experiment in the laboratory was executed to determine the actual amount of aluminium sulphate (Al_2_(SO_4_)_3_) needed to reach the targeted soil pH level. Hundred grams of air-dried soil was mixed with five incremental rates (0.1, 0.2, 0.3, 0.4 and 0.5 g) of aluminium sulphate (Al_2_(SO_4_)_3_). The soil was then moistened with distilled water at a field capacity of 60% and placed under polyethylene cover containing a hole. After 2 weeks, soil pH was measured (Baquy et al., 2017). The Al concentration in the soils of T1, T2 and T3 were 3622, 3395 and 3210 ppm, respectively. Every treatment combination was replicated five times and arranged in a Completely Randomized Design (CRD) design. The experiment was conducted for three months.

### Soil pH and electrical conductivity

Both the soil pH and electrical conductivity (EC) were recorded at the start and end of the experiment from five replicates. The soil sample was first oven-dried at 60 °C for 24 h and then air dried before sieving through a 2 mm mesh. The soil solution was made up of 1:2.5 of soil to deionized water, shaken and then left to stand for an hour for the determination of pH by using a pH meter with 0.01 resolution (HI211-01, Hanna Ins., US). Next, the volume of water was doubled before determining the electrical conductivity of the soil sample. The samples were then shaken mechanically at 15 rpm for about an hour to dissolve soluble salts before using a conductivity benchtop meter with automatic temperature compensation (HI-2315, Hanna Ins., US) to determine the EC.

### Plant height and plant biomass

Plant height was recorded at the beginning of the experiment and before harvest using a measuring tape. Readings were taken from five replicates. Plant biomass was recorded at harvest. Fresh weight of plants was taken by using weight balance right after the plants were harvested. The plants were then partitioned into roots and shoots and dried at 80 °C for 48 h after which the constant dry weights were recorded (Chen et al., 2013).

### Root profile

Root profile which included total root length, root volume and root diameter were analyzed using WinRHIZO (Pro v. 2008a., Regent Instruments Inc., Canada). Root diameter classes were ranged with a slight modification in order to obtain clear visibility of the root data acquired as very fine (<0.1 mm), fine (0.1–0.2 mm), medium (0.2–0.5 mm), and coarse roots (>0.5 mm) at 10-cm intervals. The Root Length Density was determined by using the following formula:

\begin{eqnarray*}& & \text{Root Length Density}= \frac{\text{Total Root Length}}{\text{Soil Volume}} . \end{eqnarray*}

### Physiological parameters

Photosynthetic rate, stomatal conductance and transpiration rate were measured by using a portable photosynthesis system (LiCor-6400; LiCor Inc., USA) between 0900 h to 1200 h with a PAR range between 1000 to 2100 µE m-2 s-1. The temperature, external carbon dioxide concentration and relative air humidity were 30  ± 2 °C, 390 µm mol −1 and 60%, respectively.

### Determination of aluminium concentration in plant shoot and soil

About 1 g of sample was weighed into a reaction vessel, after which aqua regia solution (1 HNO_3_: 3 HCl) was added into the vessel for microwave digestion (McGrath & Cunliffe, 1985). The digester block was gradually heated to 180 °C and the vessels were taken off the blocks when the samples were fully digested. The resulting extracts were then solubilized and filtered. About 1 ml of aliquots were transferred to PPE tube and diluted with 9 ml of distilled water. The Al content was then determined at 396.53 nm using ICP-OES (Perkin Elmer, Optima 5300DV). A series of standard solution was prepared according to the same protocol and read at the same wavelength.

### Statistical analysis

The data were analyzed using the statistical package SPSS 20.0 (SPSS Inc, Chicago, IL, USA). All the parameters were analyzed using two-way analysis of variance (ANOVA) test for comparing means of more than three groups while *t*-test for comparing means of two groups. The differences among treatments were determined using the Duncan’s New Multiple Range Test (DMRT) at 0.05 probability level.

## Results

### Soil pH and electrical conductivity

**Figure 1:**
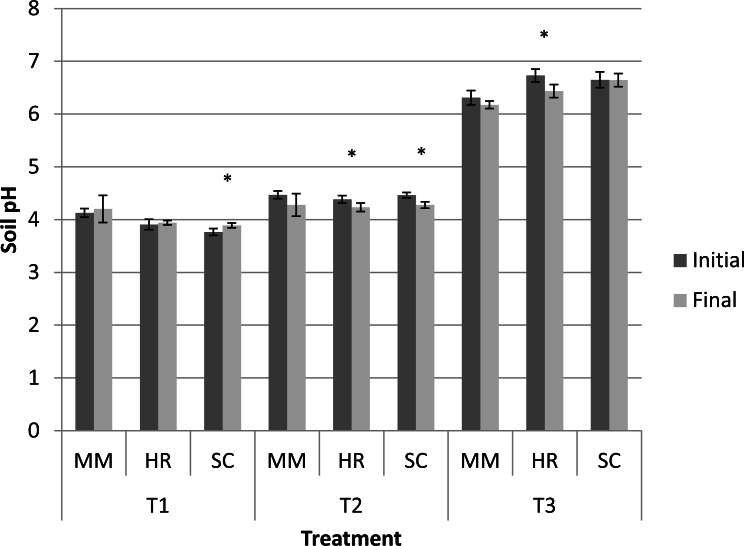
Soil pH of slope plants during initial and the end of observation. Vertical bars represent standard deviation. *T*-test was performed at *P* ≤ 0.05. *, Significant at *P* ≤ 0.05. *T*1, soil pH 3-4; *T*2, soil pH 4-5; *T*3, soil pH 6-7; MM, *M. malabathricum*; HR, *H. rosa-sinensis*; SC, *S. campanulatum*.

**Figure 2:**
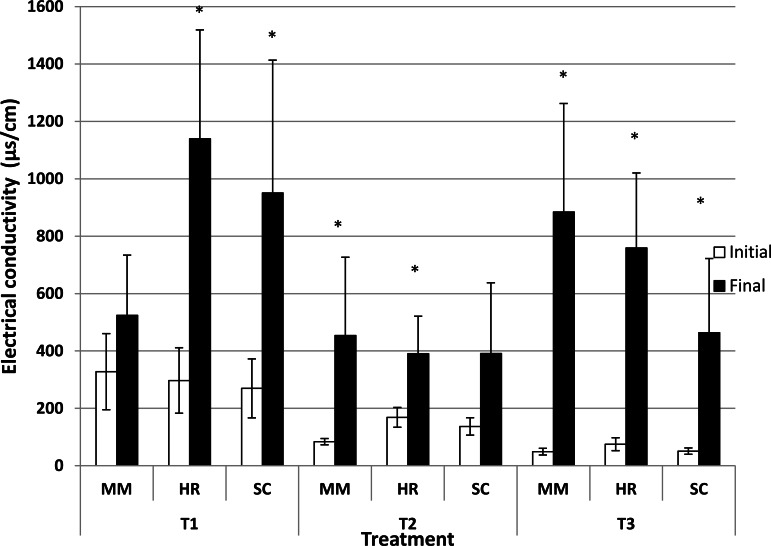
Soil pH of slope plants during initial and the end of observation. Vertical bars represent standard deviation. *T*-test was performed at *P* ≤ 0.05. *, Significant at *P* ≤ 0.05. T1, soil pH 3-4; T2, soil pH 4-5; T3, soil pH 6-7; MM, *M. malabathricum*; HR, *H. rosa-sinensis*; SC, *S. campanulatum*.

Generally, the soil pH showed minimal change from the initial observation to the final observation (Fig. 1). Nevertheless, *S. campanulatum* grown in the most acidic soil (T1) exhibited a significant increase while *H. rosa-sinensis* and *S. campanulatum* in T2 and *H. rosa-sinensis* grown in the least acidic soil (T3) displayed significant decrement in values, though the values were relatively low. Meanwhile, EC of soil solutions were not significant in terms of plant species both during the initial and final observations (Table 1). Nevertheless, the EC was significant in terms of treatment (soil pH) whereby during the initial observation, T1 displayed the highest mean followed by T2 and T3. At the end of observation though, both T1 and T3 recorded higher EC values as compared to T2. Further, interaction of factors was observed for final EC observation. Except for *M.malabathricum* in T1 and *S.campanulatum* in T2, the rest of the treatment combination exhibited significant increases in EC values (Fig. 2). The largest increment was shown by *M.malabathricum* followed by *H.rosa-sinensis* and *S.campanulatum* grown in T3.

**Table 1:** Main and interaction effects of plant species and soil pH on electrical conductivity.

Electrical Conductivity	Initial	Final
Plant Species		
*M.malabathricum*	153.67^a^	620.80^a^
*H.rosa-sinensis*	180.34^a^	762.90^a^
*S.campanulatum*	152.59^a^	601.70^a^
Pr > F	ns	ns
Soil pH		
T1	298.25^a^	871.30^a^
T2	129.94^b^	411.80^b^
T3	58.42^c^	702.20^a^
Pr > F	^***^	^***^
Species × Soil pH	ns	^*^

**Notes.**

Means followed by the same letter in the same column are not significantly different at *p* ≤ 0.05 according to Duncan’s Multiple Range test.

ns, non-significant difference at *P* > 0.05.

* significant difference at *P* ≤ 0.05.

*** significant difference *P* ≤ 0.001.

### Height

**Figure 3:**
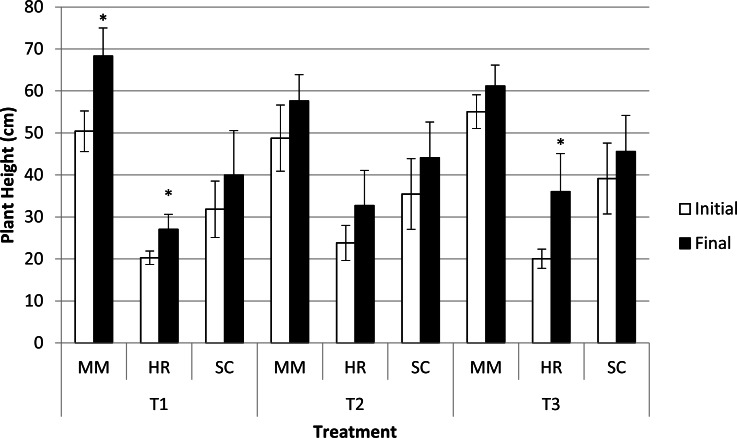
Plant height of slope plants grown in differing soil pH during initial and the end of observation. Vertical bars represent standard deviation. *T*-test was performed at *P* ≤ 0.05. *, Significant at *P* ≤ 0.05. T1, soil pH 3-4; T2 soil pH 4-5; T3, soil pH 6-7; MM, *M. malabathricum*; HR, *H. rosa-sinensis*; SC, *S. campanulatum*.

Plant height was significantly different in terms of species at both the initial and final observations. *M. malabathricum* recorded the highest value followed by *S. campanulatum* and *H. rosa-sinensis*. Besides, three combinations, namely *M. malabathricum* and *H. rosa-sinensis* grown in the most acidic soil condition and *H. rosa-sinensis* grown in T3 showed significant increment in plant height (Fig. 3).

### Root profiles

**Figure 4:**
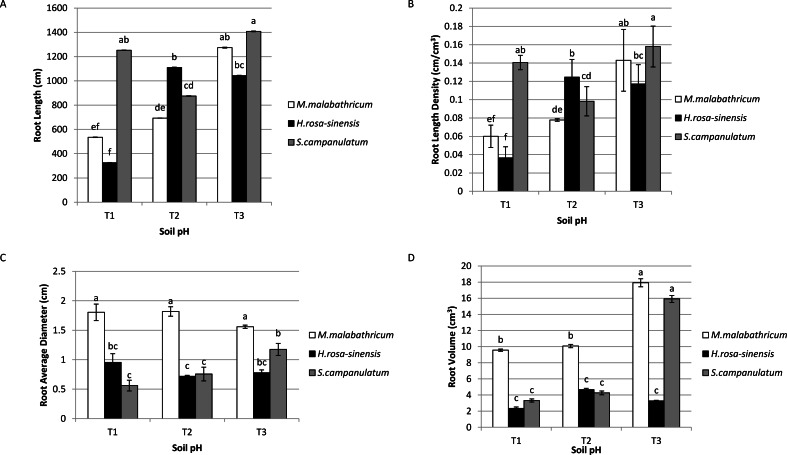
Root profiles of slope plants grown in differing soil pH. (A) Root length; (B) root length density; (C) root average diameter; (D) root volume. Vertical bars represent standard deviation. Means with same letter are not significantly different at *P* ≤ 0.05 according to Duncan’s New Multiple Range Test (DMRT). T1, soil pH 3-4; T2, soil pH 4-5; T3, soil pH 6-7.

*Syzygium campanulatum* grown in T3 showed significantly highest root length followed by *M. malabathricum* grown in the same soil condition and *S.campanulatum* grown in T1, though these three treatment combinations did not show any significant difference among them (Fig. 4A). The lowest root length was recorded by *H.rosa-sinensis* and *M.malabathricum* grown in T1. Likewise, the root length density followed the results of root length (Fig. 4B). In terms of root average diameter, *M.malabathricum* recorded the highest diameter irrespective of soil pH while *S. campanulatum* in T1 showed the lowest root average diameter, however it was not significantly different from *H.rosa-sinensis* grown in three different soil pH (Fig. 4C). In terms of root volume, *M. malabathricum* and *S. campanulatum* grown in T3 displayed the highest value followed by *M. malabathricum* grown in T1 and T2 (Fig. 4D). The rest of the treatment combinations showed a significantly lower volume though there were no significant differences amongst them.

**Figure 5:**
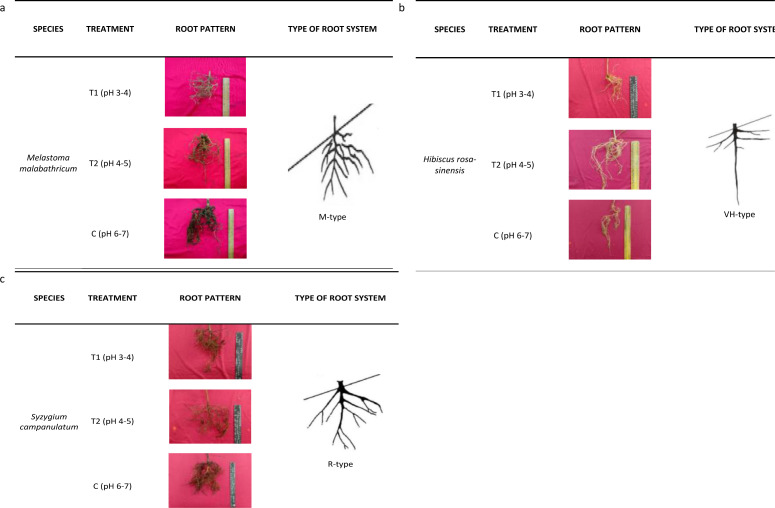
Root architecture of selected slope plants. (A) *M. malabathricum*, (B) *H. rosa-sinensis*, (C) *S. campanulatum*.

The root architecture of *M. malabathricum* showed dense and shallow roots (M-type) while *H. rosa-sinensis* displayed both lateral and tap roots which developed vertically and horizontally (Fig. 5). It was identified of having possessed the VH-type root architecture. Meanwhile, the root architecture of *S. campanulatum* was observed as oblique root (R-type).

### Plant biomass

In general, stem, root and total dry biomass were significantly different in terms of both factors whilst no interaction was observed (Table 2). As for plant species, *M.malabathricum* showed the highest root biomass followed by *H. rosa-sinensis* and *S.campanulatum*, the latter two were not significantly different. In terms of soil pH, T3 recorded the highest stem biomass followed by T2 and T1, all three were significantly different.

**Table 2:** Aluminium concentration in shoots of potential slope plants grown in differing soil pH.

Concentration of aluminium (Al) (ppm)									
Treatment	*M. malabathricum*			*H. rosa-sinensis*			*S. campanulatum*		
	T1	T2	T3	T1	T2	T3	T1	T2	T3
Initial	1322.6^b^	1485.9^a^	582.1^c^	9.8^d^	11.7^d^	6.0^d^	38.6^d^	48.8^d^	46.8^d^
Final	2044.5^a^	2029.2^a^	1205.9^b^	11.4^c^	11.9^c^	6.8^c^	47.6^c^	53.2^c^	47.1^c^

**Notes.**

Means followed by the same letter in the same column are not significantly different at *p* ≤ 0.05 according to Duncan’s Multiple Range test.

ns, non-significant difference at *P* > 0.05.

* significant difference at *P* ≤ 0.05.

** significant difference at *P* ≤ 0.01.

*** significant difference *P* ≤ 0.001.

### Physiological parameter

**Figure 6:**
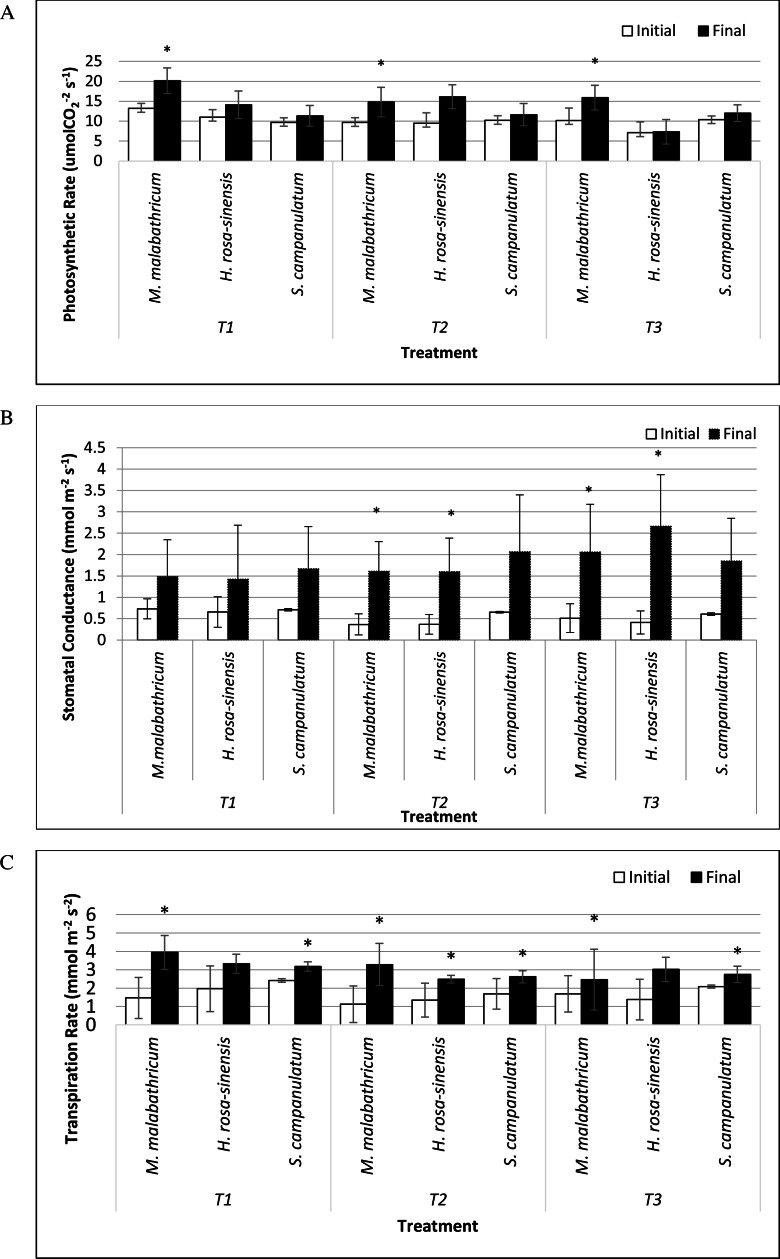
Physiological parameters of selected slope plants grown in differing soil pH during initial and the end of observation. (A) Photosynthetic rate; (B) stomatal conductance; (C) transpiration rate. Vertical bar represents standard deviation. *T*-test was performed at *P* ≤ 0.05. *, Significant at *P* ≤ 0.05. T1, soil pH 3-4; T2, soil pH 4-5; T3, soil pH 6-7.

**Figure 7:**
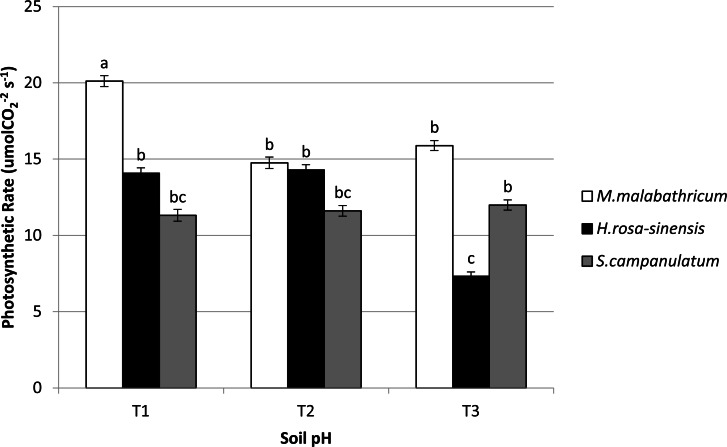
Photosynthetic rate of slope plants grown in differing soil pH. Vertical bars represent standard deviation. Means with same letter are not significantly different at *P* ≤ 0.05 according to Duncan’s New Multiple Range Test (DMRT). T1, soil pH 3-4; T2, soil pH 4-5; T3, soil pH 6-7.

*M. malabathricum* grown in the most acidic soil showed the highest photosynthetic rate followed by those grown in T3 and T2 (Figs. 6A and 7). *H. rosa-sinensis* grown in T3 showed the lowest photosynthetic rate though it was not significantly different from *S. campanulatum* grown in T1 and T2. Stomatal conductance did not show any significant result. However, in general plants grown in the least acidic soil (T3) showed comparatively higher values followed by T2 and T1. Both *M.malabathricum* and *H.rosa-sinensis* grown in T2 and T3 showed a significantly higher stomatal conductance during the final observation compared to initial observation (Fig. 6B). Transpiration rate did not show significant result, however it showed the opposite outcome of stomatal conductance. Irrespective of plant species, T1 recorded the highest rate followed by T2 and T3. Nevertheless, all treatment combination with the exception of *H. rosa-sinensis* grown in T1 and T3 recorded significant higher transpiration rate during final observation compared to initial observation (Fig. 6C). As with stomatal conductance and transpiration, water use efficiency too did not show significant result. No trend was observed for this parameter.

### Aluminium content in soil and shoot

**Figure 8:**
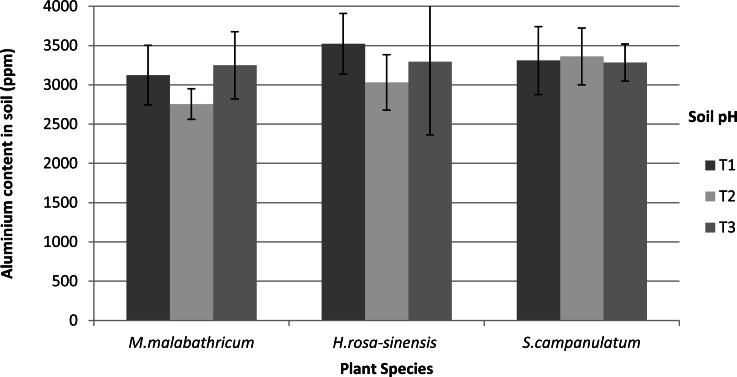
Aluminium content in soil of slope plants grown in differing soil pH two weeks before harvest. Vertical bars represent standard deviation. T1, soil pH 3-4; T2, soil pH 4-5; T3, soil pH 6-7.

Based on our results, Al content was not significantly different in the soils of differing pH. Nevertheless, *M. malabathricum* exhibited the lowest value amongst the species tested (Fig. 8). In addition, the highest decrement was shown by *M.malabathricum* grown in T2 followed by T1 at 18.8 and 13.8%, respectively. While plants grown in the acidic soils (T1 and T2) showed a reduction in Al content, T3 exhibited a slight increase of 1.2–2.3%.

The results clearly showed that *M. malabathricum* accumulated the highest Al content in its shoots with significant increment towards the end of the experiment (Table 3). The Al content of *M. malabathricum* grown in the least acidic soil (T3) doubled at the end of experiment while T1 and T2recorded 54.6 and 36.6% increases, respectively. This was followed by *S. campanulatum* and *H. rosa-sinensis* which did not show much increase in Al content.

## Discussion

The findings of the current study strongly suggest that *M. malabathricum* was adaptive to acidic soil as it showed the highest plant height, photosynthetic rate and transpiration rate in severe soil pH condition (T1). This fact is proven, for the plant species accumulated the highest percentage of Al in its shoots compared to the other two plants. According to Ma et al. (1997), plants with high tolerance to Al are better in biomass production as compared to Al-sensitive plants. Besides, a tall plant is able to capture and utilize the light more efficiently, thus enhancing assimilation process of the plant. As for root profile, *S. campanulatum* consistently recorded high root length and root length density across all three types of soil pH while *M. malabathricum* showed progressive increase in length as the soil pH increased. The latter which displayed shallow rooting may possibly have needed more time to release organic acid anions such as citrate, malate or oxalate from the root apices in response to Al-stress and thus affected root elongation (Prasetyo, 2007).

Although Al is often classified as toxic, its impact on plant growth highly depends on its concentration which differs according to genotype within the same species, physiological age, growth conditions and the length of exposure to the metal (Bojórquez-Quintal et al., 2017). Since *M. malabathricum* is an Al-accumulator, it did accumulate more than 1000 ppm of Al in its shoot while *S.campanulatum* and *H.rosa-sinensis* were non-acccumulators as the shoots recorded around 70 and 10 ppm, respectively. Previous studies suggest varying root response in relation to Al concentration in the soils from root growth stimulation at high concentration to inhibition at low concentrations and vice-versa or unaffected at any concentrations (Barcelo & Poschenrieder, 2002; Zhou et al., 2011). This testifies the differences in response mechanism at the whole-plant level as well as cellular and tissue levels.

In spite of the toxic effect of Al on plant growth, there are reports of beneficial effect of the element on plants. According to Pilon-Smits et al. (2009) plants adapted to acid soils show growth stimulation when exposed to Al. Both *S. campanulatum* and *M. malabathricum* might have benefited from the presence of Al in the soil via root induction.

Interestingly, as *M. malabathricum* is a hyperaccumulator of Al (Osaki, Watanabe & Tadano, 1997; Ghanati, Morita & Yokota, 2005; Tomioka, Oda & Takenaka, 2005), the metal is essential for its growth as evident by the white coloration and elongation of roots that have also been reported in *M. malabathricum* in presence of Al (Oda, 2003; Ghanati, Morita & Yokota, 2005; Watanabe, Jansen & Osaki, 2006; Schmitt et al., 2016). On the other hand, its absence or insufficiency had led to chlorosis, morphological changes and leaf curling (Watanabe, Jansen & Osaki, 2006) besides secreting mucilage in the roots to accumulate Al in soils (Watanabe, Jansen & Osaki, 2005; Watanabe et al., 2008a; Watanabe et al., 2008b).

**Table 3:** Aluminium concentration in shoots of potential slope plants grown in differing soil pH.

Concentration of aluminium (Al) (ppm)									
Treatment	*M. malabathricum*			*H. rosa-sinensis*			*S. campanulatum*		
	T1	T2	T3	T1	T2	T3	T1	T2	T3
Initial	1322.6^b^	1485.9^a^	582.1^c^	9.8^d^	11.7^d^	6.0^d^	38.6^d^	48.8^d^	46.8^d^
Final	2044.5^a^	2029.2^a^	1205.9^b^	11.4^c^	11.9^c^	6.8^c^	47.6^c^	53.2^c^	47.1^c^

**Notes.**

Means followed by the same letter in the same row are not significantly different at *p* ≤ 0.05 according to Duncan’s Multiple Range test.

Meanwhile, according to White et al. (2013) high root density corresponds to higher production of lateral roots and root hairs whereas Normaniza & Barakbah (2006) observed the highest root length density in a stable slope due to the high density of vegetation which resulted in lower water content (SWC). Besides, root length density was positively correlated to shear strength while SWC was negatively related to both soil penetrability and shear strength. On the other hand, Bengough et al. (2005) found that decreased root elongation was corroborated to reduction in production rate and axial cell elongation. Also, root average diameter of *M. malabathricum* outperformed the other two plant species irrespective of soil pH. Similarly, *M. malabathricum* showed increment in root volume. Root thickening has been observed for roots that were exposed to elevated soil acidity that may be due to the widening of cells, which permits greater pressure to be applied on the soil (Croser, Bengough & Pritchard, 2000; Bengough et al., 2006). *Melastoma malabathricum* showed favourable characteristic of soil reinforcement as high root volume can elevate the soil root matrix which will eventually increase the effectiveness in absorbing moisture, thus improving matric suction (Saifuddin, Osman & Rahman, 2013). On the contrary, the poor root growth of *H. rosa-sinensis* in comparison to the other two species could be due to its ‘non-accumulator’ of Al status. The acidic soil might have moderately affected the division of root cells and the ability of the root to elongate which leads to reduced root growth and branching (Gazey & Davies, 2009). Further, low pH facilitates the influx of acid cation such as hydrogen ion into root tissues which damages the root membranes (Iqbal, 2012). The influx also depolarises the plasma membrane potential and causes cytoplasmic acidification (Babourina et al., 2001). In the current study, *M. malabathricum* did not display any symptoms of stunted root growth even under severe acidic condition, while exhibiting four-fold increase in root volume compared to the other plants. This may be due to the plant’s adaptive nature of Al toxicity.

Consequently, the root architecture in this study was classified according to Yen (1987) who proposed a root system based on the tap, lateral and horizontal roots, classifying them into five types, namely, H, M, R, V and VH. The H- and VH-root types were deemed suitable for soil reinforcement, slope protection and wind resistance. On the other hand, the M-type was effective in controlling soil erosion while the V-type was suitable for wind resistant (Reubens et al., 2007). R-type root architecture is favourable in protecting slope from failure and was found to be more effective than V-type root in improving soil shear strength (Fan & Chen, 2010). Based on this study, the root systems of *M.malabathricum*, *H.rosa-sinensis* and *S.campanulatum* were identified as M, VH- and R-types, respectively. Irrespective of the differences in root profile parameters, the root architecture of the slope plant species was not affected by soil acidity. Moreover, unlike the norm, where acidic soil retards the growth and development of plants, our observation implies the selected plant species as having tolerance mechanism towards acidic condition for they did not suffer from root growth inhibition. Thus, we suggest *M.malabathricum* which possessed dense and shallow roots to be planted at the toe or top of the slope (Ghestem et al., 2014) while *H.rosa-sinensis* and *S.campanulatum* to be planted in the middle of a slope. *Melastoma malabathricum* lacked taproot, but possessed a highly fibrous shallow rooting near the soil surface that makes it unsuitable for planting in the middle of a slope (Saifuddin & Normaniza, 2016). *Hibiscus rosa-sinensis* was identified as more promising than the other root systems because it had homogenous reinforcement effect on both lateral and tap roots which developed vertically and horizontally while *S. campanulatum* which had oblique root may withstand soil catastrophe (Li et al., 2016).

In addition, we found that *M. malabathricum* exhibited the highest dry biomass which was in line with Ma et al. (1997) who reported that Al-tolerant plants recorded better dry weight performance than Al-sensitive plants. On the contrary, *H. rosa-sinensis* experienced inhibition of root elongation for it recorded the lowest root biomass in T1 and T2. The plant species is postulated to be Al-sensitive thus the reduced total biomass.

In terms of photosynthetic rate, *M. malabathricum* recorded significant increases across all soil pH levels. In addition, the species showed the highest photosynthetic rate when grown in the most acidic soil (T1) during the final observation. However, no significant differences obtained for stomatal conductance and transpiration at both initial and final observations. Nevertheless, the stomatal conductance of *M. malabathricum* and *H. rosa-sinensis* showed significant increments in T2 and T3 as compared to initial observation. On the other hand, no clear trend was observed for transpiration rate. Although previous study by Evans (2000) allegedly proved that Al have adverse impact on photosynthetic rate due to Al toxicity, *M. malabathricum,* however, showed a tolerance mechanism towards acidic soil for its well established roots led to high water absorption capacity thus increasing the rate of photosynthesis. Besides aiding in water absorption, these extensive roots might have aided in the uptake of potassium which is essential for chlorophyll synthesis that subsequently led to a higher rate of photosynthesis (Ainsworth & Rogers, 2007). As for transpiration rate, though insignificant, *M. malabathricum* showed the highest transpiration rate when grown in T1 and T2, a pivotal characteristic of slope plant species. A slope that is drier will enhance slope stability as a result of reduced water saturation level (Saifuddin et al., 2016). Our findings clearly showed *M.malabathricum* as having considerable potential to be planted on the slopes possessing acidic soil.

## Conclusions

For the successful application of vegetations to control erosion and improve slope stability, it is vital to look into both the soil and plant properties. The understanding of the interaction of plant, soil and water is the key to address slope failures. Soil bioengineering makes use of vegetation to enhance root reinforcement in terms of mechanical and hydrological aspects. However, not all plants are adaptive to low soil pH which is the case with slope soil. *Melastoma malabathricum* exhibited better rooting, increased biomass production and higher photosynthetic rate in comparison to *H. rosa-sinensis* and *S. campanulatum*. As such, we propose *M. malabathricum* to be grown on slopes featuring acidic soil as a form of soil rehabilitation.

##  Supplemental Information

10.7717/peerj.9595/supp-1 Dataset S1 Raw data

## Additional Information and Declarations

### Data Availability

The following information was supplied regarding data availability: Raw data is available in the Supplementary Files.
